# Fish oil consumption prevents glucose intolerance and hypercorticosteronemy in footshock-stressed rats

**DOI:** 10.1186/1476-511X-10-71

**Published:** 2011-05-11

**Authors:** Ricardo Eguchi, Flavia R Scarmagnani, Claudio A Cunha, Gabriel IH Souza, Luciana P Pisani, Eliane B Ribeiro, Claudia M Oller do Nascimento, Regina C Spadari-Bratfisch, Lila M Oyama

**Affiliations:** 1 Departamento de Fisiologia. Universidade de São Paulo - UNIFESP - Rua Botucatu, 862, 2nd floor, Edifício de Ciências Biomédicas. Vila Clementino, São Paulo, SP, Brazil; 2 Departamento de Biociências. Universidade Federal de São Paulo - UNIFESP - Av. Ana Costa, 95. Vila Mathias, Santos, SP, Brazil

## Abstract

**Background:**

Environmental stress plays an important role in the development of glucose intolerance influencing lipid and glucose metabolism through sympathetic nervous system, cytokines and hormones such as glucocorticoids, catecholamines and glucagon. Otherwise, fish oil prevents glucose intolerance and insulin resistance. Although the mechanisms involved are not fully understood, it is known that sympathetic and HPA responses are blunted and catecholamines and glucocorticoids concentrations can be modulated by fish consumption. The aim of the present study was to evaluate whether fish oil, on a normal lipidic diet: 1) could prevent the effect of footshock-stress on the development of glucose intolerance; 2) modified adiponectin receptor and serum concentration; and 3) also modified TNF-α, IL-6 and interleukin-10 (IL-10) levels in adipose tissue and liver. The study was performed in thirty day-old male Wistar randomly assigned into four groups: no stressed (C) and stressed (CS) rats fed with control diet, and no stressed (F) and stressed (FS) rats fed with a fish oil rich diet. The stress was performed as a three daily footshock stress sessions.

**Results:**

Body weight, carcass fat and protein content were not different among groups. FS presented a reduction on the relative weight of RET. Basal serum glucose levels were higher in CS and FS but 15 min after glucose load just CS remained with higher levels than other groups. Serum corticosterone concentration was increased in CS, this effect was inhibited in FS. However, 15 min after footshock-stress, corticosterone levels were similar among groups. IL-6 was increased in EPI of CS but fish oil consumption prevented IL-6 increase in FS. Similar levels of TNF-α and IL-10 in RET, EPI, and liver were observed among groups. Adipo R1 protein concentration was not different among groups. Footshock-stress did not modify AdipoR2 concentration, but fish oil diet increases AdipoR2 protein concentration.

**Conclusions:**

Footshock-stress promotes glucose intolerance associated to corticosterone serum level and epididymal white adipose tissue IL-6 concentration increase. The fish oil consumption by stressed rats normalized the stress responses. These results suggested that fish oil intake could be useful to minimize or prevent the development of diseases associated to the stress.

## Background

Among environmental factors, stress plays an important role in the induction of glucose intolerance. There are two major components of the stress response: the autonomic nervous system and the hypothalamic-pituitary-adrenal axis (HPA) [1].

The regulation of glucose and lipid metabolism is under the influence of the sympathetic nervous system, cytokines [2], and increased plasmatic levels of glucocorticoids and glucagon acting synergistically with the catecholamines to increase glycemia during stress [3]. Corticosterone, in rodents, is a hormone released by adrenal cortex after the activation of HPA axis [1] and increases glucose production by the liver and decreased peripheral glucose transport and utilization [4].

Glucocorticoids cause a decrease in adipocyte gene expression and secretion of adiponectin and interleukin-6 (IL- 6) [5,6]. On the other hand, in primary cultures of murine adipocytes, norepinephrine, isoprenaline, and a β3-selective agonist have been shown to stimulate IL-6 gene expression and protein secretion [6].

Moreover, pro-inflammatory adipokines, such as tumor necrosis factor- α (TNF-α) and IL-6 lead to the development of insulin resistance [7,8]. Conversely, adiponectin (anti-inflammatory adipokine), binding to its receptors expressed in fat, liver and muscle tissues, activates several intracellular signaling pathways and improves insulin sensitivity, promoting glucose utilization, increasing fatty acid oxidation and, glucose uptake in the muscle, and reducing gluconeogenesis in the liver [9].

Previously, we have shown that three daily sessions of foot-shock stress in rat caused an increase in the plasma corticosterone, insulin and glucose levels accompanied by insulin resistance in isolated white adipocytes and soleus muscle. These results demonstrated that foot-shock stress could be a useful model for studying glucose intolerance and insulin resistance [10,11].

Several studies have been shown that fish oil could prevent glucose intolerance and insulin resistance [12,13]. It has been demonstrated in both human [14-16] and animals models [17-19] the beneficial anti-stress effects of fish oil on stress response. The mechanisms by which fish oil consumption exerts the anti-stress effect are not fully understood. But it is known that sympathetic and HPA response are blunted [14] and fish oil can modulate catecholamines and glucocorticoids concentrations [15,16].

The majority of the research, about the effects of fish oil on glucose tolerance and insulin resistance, utilized a high fat diet or additional supplementation. In these sense, the aim of the present study was to evaluate whether fish oil, on a normal lipidic diet: 1) could prevent the effect of footshock-stress on the development of glucose intolerance; 2) modified adiponectin receptor and serum concentration; and 3) also modified TNF-α, IL-6 and interleukin-10 (IL-10) levels in adipose tissue and liver. These results could contribute to identify whether a normal lipidic diet rich in n-3 polyunsaturated fatty acid ameliorate the effects of stress on adipokines and on glucose intolerance.

## Materials and methods

### Animal care

All procedures for the care of the animals used in this study were previously approved by the Experimental Research Committee of the Federal University of São Paulo (protocol n°2007/1346). Twenty-four male Wistar rats (*Rattus norvegicus*) were housed collectively (4 rats/cage) after weaning until 93 d of life in a light (12 h light/dark cycle) and temperature (24 ± 1°C) controlled room. Diet and water were available *ad libitum*. Body weight and food intake were recorded weekly.

Animals were randomly divided into 4 experimental groups: Soybean Non stressed **(C)**, received a diet containing soybean oil as a source of lipids; Fish Oil Non stressed **(F)**, received a diet containing fish oil as a source of lipids; Soybean Stressed **(CS)**, received a diet containing soybean oil as fat source and were submitted, in the 91st to 93rd days of life, to the footshock-stress; and Fish Oil Stressed **(FS)**, received a diet containing fish oil as fat source and were submitted to the footshock-stress sessions.

### Diet composition

After weaning until 60 d of life the rats were feed with growth diet (AIN-93G), containing 20% protein and 8% lipids, as described by American Institute of Nutrition (AIN-93) [20]. After 60 d of life, until 93 d of life rats were fed with a maintenance diet (AIN -93M), with 5% lipids and 14% protein. C and CS diet contained soybean oil (Liza^®^, Itumbiara-GO, Brazil) as lipid source. While F and FS diet contained fish oil as lipid source (Campestre^®^, São Bernardo do Campo-SP, Brazil).

### Stress Procedure

Each rat underwent three daily sessions of unsignaled, inescapable footshocks. The animals were placed in a Plexiglas chamber (26 × 21 × 26 cm) provided with a grid floor made of stainless-steel rods (0.3 cm in diameter and spaced 1.0 cm apart). During the 30 min sessions, which occurred between 8:00 and 10:30 am, the footshocks were delivered by a constant current source controlled by a microprocessor, with constant intensity of 1.0 mA, with a duration of 1 s at random intervals of 5-25 s (mean interval of 15 s) [11]. In the first and second sessions of stress the rats were at the fed state. At the third session of stress, rats were fasted for 12 h to perform the Oral Glucose Tolerance Test (OGTT) after the last session of stress.

### Oral glucose tolerance test (OGTT)

After 12 h fasting, body weight recording and the third footshock-stress session for the stressed rats (CS and FS), all groups were submitted to OGTT. The test started with collection of blood samples through a small incision in tail to determine baseline parameters. Glucose solution (50% glucose dissolved in saline 0.9%) was administered by intragastric gavage (2 mL/kg). Blood samples were collected at 15, 30, 45, 60 and 90 minutes after glucose load. Throughout the procedure animals were awake and free moving in the cage. To calculate area under the curve (AUC) we used Matthews et al. [21] formula's: ; where "t" means time and "y" concentration.

### Biochemical and Hormonal serum analysis

Blood samples collected after the last stress session and before the glucose load administered in the OGTT was used to evaluate the baseline blood glucose, adiponectin and insulin concentration. Serum Insulin (R&D Systems^®^, Minneapolis-MN, USA), adiponectin (R&D Systems^®^, Minneapolis-MN, USA) and corticosterone (Stressgen^®^, Ann Harbor-MI, USA) concentrations and TNF-α, IL-6 and IL-10 (R&D Systems^®^, Minneapolis-MN, USA) in adipose tissue and liver levels were determined by enzyme-linked immunosorbent assay (ELISA). Glucose was determined with the commercially available kit PAP Liquiform Glucose (Labtest^®^, Lagoa Santa-MG, Brazil). Protein was quantified with the BCA Reagent for proteins - Lowry Modified (BioAgency^®^, São Paulo-SP, Brazil).

### Carcass lipid and protein content

Carcasses were eviscerated, weighed, and stored at -20°C. Lipid content was measured as described by Stansbie et al. [22] and standardized using the method described by do Nascimento and Williamson [23]. Briefly, the eviscerated carcass was autoclaved at 120°C for 90 min and homogenized with double the mass of water. Triplicate aliquots of this homogenate were weighed and digested in 3 ml of 30% KOH and 3 ml of ethanol for at least 2 h at 70°C in capped tubes. After cooling, 2 ml of 12 N H_2_SO_4 _were added and the sample was washed three times with petroleum ether for lipid extraction. Results are expressed as grams of lipid per 100 g of carcass. For protein measurements, aliquots of the same homogenate were heated to 37°C for 1 hour in 0.6 N KOH with constant shaking. After clarification by centrifugation, protein content was quantified.

### Western blotting procedures

Animals were decapitated. Retroperitoneal (RET) and epididymal (EPI) adipose tissues and liver were removed and homogenized in ice-cold solubilization and total protein extraction buffer (100 mM Tris, pH 7.5, 10 mM ethylene acetic acid, 100 mM sodium fluoride, 10 mM sodium orthovanadate, 2 mM phenylmethylsulfonyl fluoride, 10 mM sodium pyrophosphate and 0.1 mg/mL aprotinin. After homogenization, Triton X-100 was added, to a final concentration of 1%. Samples rested in ice for 30 min and were clarified by centrifugation. Equal amounts of protein (30 μg for liver and 120 μg for RET and EPI) were loaded in 10% sodium dodecylsulfate polyacrylamide gel electrophoresis, electrophoretically separated, and transferred to nitrocellulose membranes. Membranes were incubated with the appropriate primary antibody: AdipoR1 (SC-46748), AdipoR2 (SC-46751), HSD1 (SC-20175) and α-tubulin (SC-58664) (Santa Cruz Biotechnology^®^, Santa Cruz-CA, USA). After secondary antibody incubation, detection was performed by chemiluminescence with an enhanced chemiluminescence reagent (Amersham Biosciences^®^, Piscataway-NJ, USA). Membranes were stripped and reblotted with α-tubulin. Quantitative analyses were performed with ImageJ^® ^software and the results as standardized with α-tubulin as load control. Data are expressed as percentage (%) of control.

### Data analysis

The results are expressed as mean ± SEM. For multiple comparisons of means was performed analysis of variance (ANOVA) two-way with subsequent use of the Tukey *post-hoc *test. Statistical significance was set at *P <*0.05.

## Results

### General characteristics of the experimental groups

Body weight, carcass fat and protein content (Table 1) and food intake (data not showed) were similar among all groups.

**Table 1:** Animal characteristics from body composition, serum basal levels of adiponectin, insulin, glucose and corticosterone of Control (C), Stressed (CS), Fish Oil (F) and Fish oil Stressed (FS) groups

	C	CS	F	FS	*p*
**Initial body weight (g)**	94.6 ± 3.4	94.6 ± 5.1	91.9 ± 3.9	92.2 ± 3.2	ns
**Final body weight (g)**	369.2 ± 9.3	373.3 ± 10.9	376.9 ± 9.5	361.6 ± 10.2	ns
*Carcass content*					
**Protein (g/100 g)**	16.3 ± 1.6	16.9 ± 0.9	17.1 ± 1.8	18.9 ± 1.5	ns
**Fat (g/100 g)**	8.6 ± 1.0	8.8 ± 1.2	7.7 ± 1.2	8.0 ± 1.1	ns
*Relative weight*					
**RET (g/100 g)**	2.00 ± 0.17	1.54 ± 0.15	1.48 ± 0.09	1.24 ± 0.17*	0.05
**EPI (g/100 g)**	2.12 ± 0.20	1.97 ± 0.18	1.71 ± 0.26	1.43 ± 0.11	ns
**LIV (g/100 g)**	2.88 ± 0.05	2.83 ± 0.13	2.91 ± 0.08	2.65 ± 0.03	ns
**Adiponectin (mg/mL)**	7.34 ± 0.56	8.04 ± 0.71	7.57 ± 0.54	7.49 ± 0.58	ns
**Insulin (ng/mL)**	1.01 ± 0.37	0.96 ± 0.29	0.75 ± 0.16	0.67 ± 0.21	ns
**Glucose (mg/dL)**	95.1 ± 3.4	133.4 ± 10.1§	93.5 ± 3.0	125.19 ± 6.0§	0.0001
**Corticosterone (ng/mL)**	182.0 ± 23.5	376.1 ± 40.1*	122.2 ± 9.1	108.8 ± 42.7	0.04

Data are means ± SEM. N = 6 rats/group. * Different from Control (C) group *p < 0.05*. § Different from Control (C) and Fish Oil (F) groups *p < 0.05*.

Neither footshock-stress nor fish oil consumption altered relative weight of RET, EPI and liver. However, the association of fish oil consumption and footshock-stress reduced the relative weight of RET in relation to C, with no differences in EPI and liver (Table 1).

### OGTT and Biochemical and Hormonal serum analysis

**Figure 1:**
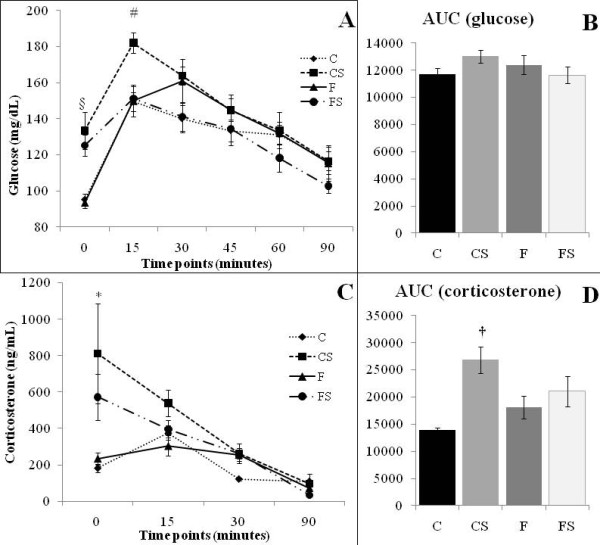
**OGTT-Oral Glucose Tolerance Test (A), glucose AUC (B) serum corticosterone levels (C) and corticosterone AUC (D) of Control (C), Stressed (CS), Fish Oil (F) and Fish oil Stressed (FS) groups**. Data are means ± SEM. N = 6 rats/group. * Different from Control (C) group *p < 0.05*. † Stressed rats (CS) different from non stressed rats (C and F). § Footshock-stressed rats (CS and FS) different from non stressed rats (C and F) *p < 0.05*. # Different from all other groups *p < 0.05*.

Basal serum glucose levels were higher in stressed (CS and FS) than in non stressed (C and F) animals. However, 15 min after glucose load, CS serum glucose levels remained elevated compared to other groups, but fish oil ingestion abolished the effect of footshock-stress on glucose levels (Figure 1A). Conversely, glucose AUC was similar among groups (Figure 1B).

Insulin and adiponectin serum concentration were similar among groups (Table 1).

Immediately after footshock-stress, serum corticosterone concentration was increased in CS, but fish oil diet inhibits this effect (FS) (Table 1). 15 min after footshock-stress session, corticosterone levels were similar among groups (Figure 1C). Still, CS corticosterone AUC was higher than the others groups (C, F and FS) (Figure 1D).

### IL-6, TNF-α. and IL-10 levels in RET, EPI and liver

**Figure 2:**
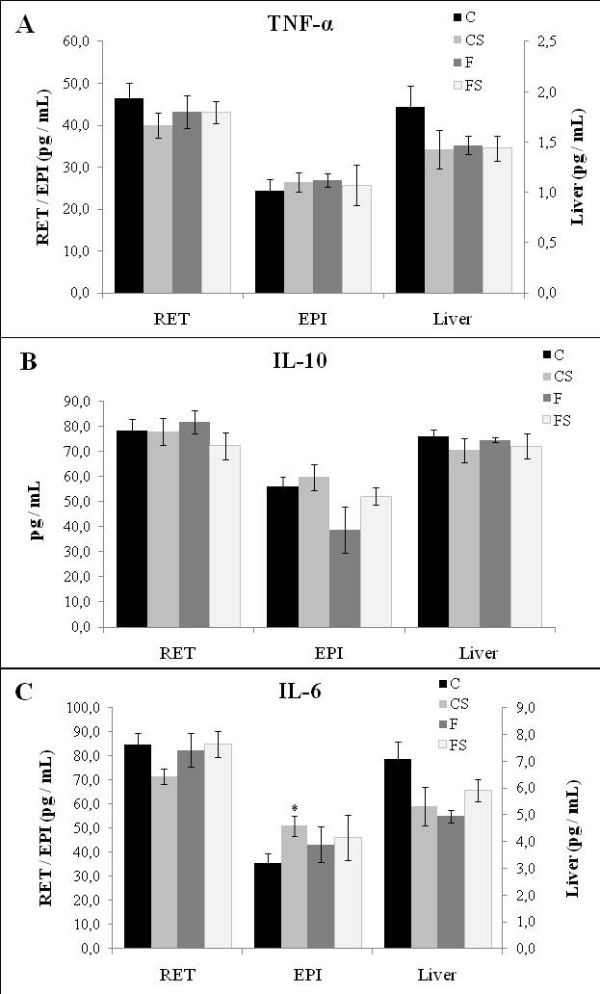
**TNF-α (A), IL-10 (B) and, IL-6 (C) protein contents in retroperitoneal (RET), epididymal (EPI) adipose tissues and liver of Control (C), Stressed (CS), Fish Oil (F) and Fish oil Stressed (FS) groups**. Data are means ± SEM. N = 6 rats/group. * Different from Control (C) group *p < 0.05*.

Footshock-stress (CS) lead to an increase of IL-6 levels in EPI compared to C. Fish oil consumption (F) did not alter IL-6 levels and prevented IL-6 increase in footshock-stressed animals (FS) (figure 2C). Similar levels of TNF-α and IL-10 in RET, EPI, and liver were observed among groups (figure 2A, B).

### AdipoR1/R2 quantity in adipose tissue and liver

**Figure 3:**
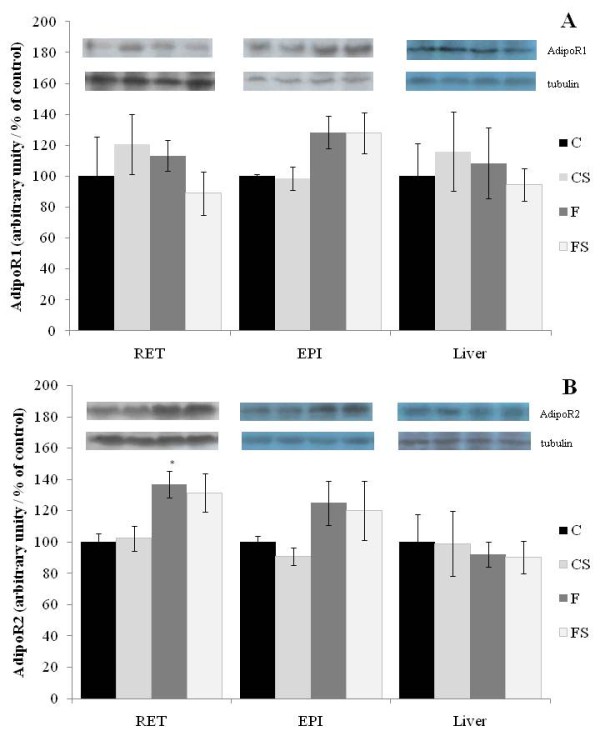
**AdipoR1(A) and AdipoR2 (B) protein contents in retroperitoneal (RET), epididymal (EPI) adipose tissues and liver of Control (C), Stressed (CS), Fish Oil (F) and Fish oil Stressed (FS) groups**. Results were quantified by densitometry and α-tubulin was used as load control. Data are expressed as % of control (C) and are means ± SEM. N = 6 rats/group. * Different from Control (C) group *p < 0.05*.

Adipo R1 protein concentration was similar in the study tissues among groups (Figure 3A). The footshock-stress did not modify AdipoR2 concentration in studied groups. Although, fish oil diet increases AdipoR2 protein concentration in RET as compared to control diet (F vs. C) (Figure 3B).

## Discussion

The present study shows that footshock-stress caused a glucose intolerance accompanied by an increase in corticosterone level and IL-6 epididymal protein content. The fish oil consumption blunted these effects of stress, demonstrating the preventive effect of fish oil consumption in development of glucose intolerance promoted by footshock-stress.

The final body weight and carcasses lipid and protein content were not modified by 3 days of footshock-stress and/or fish oil consumption for 8 weeks. Previously, Papakonstantinou et al. [24], using a repeated restraint stress for 3 days, 3 hours per day, showed that consumption of hyperlipidic diet rich with fish oil (2 weeks before stress and 2 after) did not ameliorate stress-induced anorexia and weight loss. Similar results was observed in rats treated with normolipidic diet containing fish oil for 45 days submitted for 96 hours of sleep deprivation [25]. Taken these results all together it could be suggested that the duration of fish oil ingestion and type of stressor could influence the effect of stress in body weight.

However, in the present study the footshock-stress in fish oil treated rats caused a decrease in RET weight. Fish oil reduces fatty acid accumulation [26,27] and prevents diet induced obesity [28], mainly through the regulation of lipid metabolism by inhibiting lipogenesis, promoting lipolysis and fatty acid oxidation, and suppressing preadipocyte differentiation [29].

As previously reported [10,11] footshock-stress caused a glucose intolerance and increase corticosterone serum levels, in addition in the present study we demonstrated that the ingestion for 8 weeks of normolipidic diet rich in fish oil prevented these effects.

Insulin resistance promoted by glucocorticoid has long been known and is associated with increased hepatic glucose production and decreased peripheral glucose transport and utilization [4]. In the present study, CS exhibited a marked increase in corticosterone concentrations in relation to all other groups, including FS, which reflects OGTT response. This result suggests that glucose intolerance promoted by footshock occurred in response to activation of HPA axis, and that consumption of fish oil blunted this effect, protecting against glucose intolerance. This result underscores the anti-stress effect of fish oil consumption.

Based on these find, we seek to establish whether the effect of fish oil was not only related to the normal corticosterone level observed in FS group, but also if it could be associated to fish oil effects on adipokines related to glucose intolerance.

The increase in adipoR2 in fish oil fed rats might be involved in this response, but further investigations will be required to elucidate the mechanisms involved.

The insulin-sensitizing adipokine adiponectin is another important factor involved in glucose intolerance [30,31]. Adiponectin is specifically expressed in adipose tissue and has antiatherogenic and antidiabetic properties [31-33] and its effects are mediated by adiponectin receptors, adipoR1 and adipoR2 [32]. AdipoR1 is abundantly expressed in skeletal muscle, whereas adipoR2 is predominantly found in liver [31,32] and both are also expressed in adipose tissue [32].

The effects of stress and glucocorticoids on adiponectin regulation are controversial. In human adipocytes, dexamethasone inhibits adiponectin release [34]. In human volunteers treated with dexamethasone plasma adiponectin levels were unchanged [35], while in another study a small rise was found [36] but adiponectin was decreased in subjects treated with hydrocortisone [37]. Catecholamine also modulates adiponectin, β-adrenergic stimulation downregulate adiponectin gene expression [30,38] but upregulates AdipoR2 expression [30]. These data suggest that fish oil consumption, although not alter serum levels of adiponectin, may have improved the sensitivity to this hormone contributing to the protective effect of fish oil on glucose intolerance induced by stress.

Increase in pro-inflammatory adipokines, such as TNF-α and IL-6, also leads to glucose intolerance and insulin resistance [7].

The fish oil and stress have been reported to exert pro and anti-inflammatory effects [29,39]. In these sense, we decided to measured the retroperitoneal and epididymal white adipose tissue and liver TNF-α, IL-6 and IL-10 protein content.

Neither footshock-stress, fish oil consumption nor the interaction between fish oil and footshock-stress caused modification on TNF-α and IL-10 protein content in the studied tissues. However, the footshock-stress increased IL-6 protein concentration in EPI and fish oil consumption impaired this effect.

Glucocorticoids cause a decrease in adipocyte gene expression and secretion of adiponectin and IL- 6 [5,6]. On the other hand, in primary cultures of murine adipocytes, norepinephrine, isoprenaline, and a β3-selective agonist have been shown to stimulate IL-6 gene expression and protein secretion [6]. Conversely, fish oil rich diet decreased plasma levels of norepinephrine in healthy subjects [40]. In the present study, the decrease in EPI IL-6 protein content in FS group is not related to an increase in glucocorticoid, since stress did not modified this hormone concentration in fish oil treated rats. However, taken our results with the literature ones, the decrease in IL-6 could be related to a decrease in norepinephrine caused by fish oil diet in stressed rats (FS). The catecholamines were not evaluated, which is a limiting factor in this study, since these hormones, like corticosterone, reflect the stress response and affects many metabolic effects. However the measurement of serum corticosterone demonstrated its important role in the footshock-stress and fish oil consumption response.

Adipose tissue IL-6 production may account for about 25% of the circulating IL-6 [41]. Then, the decrease in IL-6 in EPI of FS comparing to CS reflect, probably, in the serum IL-6 concentration and in the normal glucose tolerance test observed in FS rats in relation to CS rats.

In summary, footshock-stress promoted glucose intolerance associated to corticosterone serum level and epididymal white adipose tissue IL-6 concentration increase. The fish oil consumption by stressed rats normalized the stress responses. These results suggested that fish oil intake could be useful to minimize or prevent the development of diseases associated to the stress.

## Authors' contributions

RE made substantial contributions to conception and design, all the experimental analysis and acquisition of data and also analysis and interpretation of data. FRS carried out the experimental analysis and acquisition of data. CAC carried out the experimental analysis and acquisition of data. GIHS carried out the experimental analysis and acquisition of data. LPP participated in all molecular and biochemical analyzes. EBR participated in the design of the study and helped to draft the manuscript. CMON participated in the design of the study and performed the statistical analysis and helped to draft the manuscript. RCSB participated in the design of the study and helped to draft the manuscript. LMO has made substantial contributions to conception and design, analysis and interpretation of data and coordination to draft the manuscript. All authors read and approved the final manuscript.

## Competing interests

The authors declare that they have no competing interests.
